# Supplementary material for: Mapping the evidence on outcomes of childhood out-of-home care: A scoping review of reviews
Source: PLoS One. 2026 Feb 25;21(2):e0325409. doi: 10.1371/journal.pone.0325409 (PMC12935228; doi:10.1371/journal.pone.0325409)
Supplement: Attachment — Submitted filename: Response to Editorial Comments PONE-D-25-25545.docx [file pone.0325409.s005.docx]

**Response to the Editor’s comments**

**Editor’s Comments**

Thank you for submitting your manuscript to PLOS ONE. After careful consideration, we have decided that your manuscript does not meet our criteria for publication and must therefore be rejected.

Specifically:

The scoping review of reviews is outside the scope for PLOS ONE. The link you have provided to your protocol is invalid and registration status is not able to be confirmed.

**Authors’ Response**

We are grateful for your time and the opportunity to submit to PLOS ONE.

Firstly, it seems that our submission was considered out of scope not because of the topic—childhood out‑of‑home care—but rather because of our chosen methodology, namely a scoping review of reviews. However, this interpretation appears difficult to reconcile with PLOS ONE’s stated aims and the following recent examples of scoping reviews‑of‑reviews published in the journal:

- Craske et al. (2024), Pharmacist-led medication reviews: A scoping review of systematic reviews, published 06 Sep 2024
  <https://doi.org/10.1371/journal.pone.0309729>
- Breslin et al. (2024), Whole systems approaches to diet and healthy weight: A scoping review of reviews, published 13 Mar 2024
  <https://doi.org/10.1371/journal.pone.0292945>
- Hypotheses and evidence related to intense sweeteners and effects on appetite and body weight changes: A scoping review of reviews, published 18 July, 2018

<https://doi.org/10.1371/journal.pone.0199558>

If there has not been a recent change in editorial policy that excludes scoping review of reviews then we think our submission is within the scope of PLOS ONE.

Secondly, we reviewed our submission in the PLOS ONE Editorial Manager system, and the protocol link embedded in the submission proof is valid and accessible on our end. However, if the link did not work for the editorial team, we would have appreciated the opportunity to rectify this before a final decision was made. For your convenience, we have reattached the protocol link here: <https://doi.org/10.17605/OSF.IO/G7D5J>

We fully appreciate the volume of submissions the journal handles and understand editorial discretion is exercised in line with scope and scientific rigor. However, given the above, we respectfully request reconsideration if appropriate, and would be grateful for any direction you may offer on next steps.

Thank you for your attention, and we look forward to your kind response.

Warm regards,
**Richmond Opoku**
[On behalf of the authors]
