# Supplementary material for: Mapping the evidence on outcomes of childhood out-of-home care: A scoping review of reviews
Source: PLoS One. 2026 Feb 25;21(2):e0325409. doi: 10.1371/journal.pone.0325409 (PMC12935228; doi:10.1371/journal.pone.0325409)
Supplement: Attachment — Submitted filename: Authors response to reviewers.docx [file pone.0325409.s006.docx]

Dear reviewers,

We sincerely thank you for your careful reading of our manuscript and for your constructive comments. We have revised the manuscript in response to all suggestions raised. For ease of reference, we reproduce each reviewer comment below, followed immediately by our corresponding response in yellow highlighted text.

**Reviewer 1 (Editor)**

The information has potential so please reduce the redundancy between graphs and texts.

We have revised the Results section to reduce redundancy between the figures and the accompanying text. Specifically, we removed repeated numerical descriptions that are already clearly displayed in the figures and retained only concise interpretive statements in the text. The revised Results now focus on summarising overall patterns and contrasts across domains, while the detailed distributions are presented in the figures.

I find the manuscript to be a bit sketchy and it would be better to have more details as noted in the reviewers comments.

We have addressed this concern by adding further detail and clarification across the manuscript in line with the reviewers’ suggestions. Specifically, we expanded the methodological explanations, strengthened the synthesis and interpretation in the Discussion, and provided additional contextual detail where necessary. In addition, we have included a paragraph on Public involvement in the methods section and Study characteristics in the results section.

There is one statement about family being the most important place---can you clarify this statement? If a child is placed out of the home it most often suggests that the home for that child is not the best place.

We did not intend to suggest that the home environment is always the best place for every child, particularly in cases where children are placed out of home due to safety or welfare concerns. What we sought to convey is that supportive relationships with family members and peers can play a protective role for care-experienced children. We have clarified this wording to better reflect this distinction, as shown in lines 424–426 of the revised manuscript.

**Reviewer 2**

Thank you for taking the time to revise this manuscript. It is my first time reviewing them, so bear with my comments as I help you improve the manuscript before it is ready for publication. This manuscript aims to provide a bird's-eye view of the reviews that were done related to children who experienced OHC. However, please ensure you define OHC. The authors cannot assume the reader knows what it means, especially in the PLOS ONE journal, which covers a wide range of research.

We agree that a clear definition of out-of-home care (OHC) is important for readers across disciplines. We have now included a concise definition in the Background at lines 91–94 of the revised manuscript.

Now to the substantial part of the manuscript, acronyming the domains carries the risk of overloading the reader’s cognitive abilities, as they have to refresh what each domain stands for. I would recommend the authors to write the factors out instead of using the acronym, such as health and emotional well-being, instead of HEW.

We have revised the manuscript to write out the domain names in full throughout the text to improve readability and reduce cognitive load for the reader. The only exception is Figure 4, where acronyms are retained due to formatting constraints. All acronyms are clearly defined to support interpretation.

Regarding the domains, how did your team determine that the review falls under that factor? It will also be helpful to readers to know the process of coding domains to reviews, because Figure 1 initially showed more factors than the total number of reviews. The footnote does not seem sufficient to describe how the authors determined which factors to assign to the review. That will need to be clear in the method section.

We have added further detail to the Methods section to clarify how outcome domains and factor levels were assigned to each review, including how reviews could be mapped to multiple domains and factors when applicable. These additional explanations address why the frequency of domains could exceed the total number of reviews and outline the process used for coding. The clarifications are provided in lines 176–180 and 183–196 of the revised manuscript.

Table 1 should list all indicators rather than selecting example ones; this will help readers orient themselves before reading the results, rather than realizing there are many indicators when they reach Figure 3.

We have revised Table 1 to include all outcome indicators rather than a selection of examples. This change is intended to help readers orient themselves to the full scope of indicators before engaging with the Results section.

Why do Figures 4 & 5 show the inverse list of factors? Keep in mind to reduce the cognitive overload for the readers to improve the reader’s appreciation of your work. The axis is also inverse too. Pick one format and stick with it throughout all charts to minimize the cognitive overload.

To improve consistency and reduce cognitive load for readers, we have reformatted Figure 4 to match the factor ordering and axis orientation used in Figure 5 and all subsequent figures.

Checking out the protocol in OSF, why is the information in OSF not included in the manuscript, such as the background and research questions? The authors should not assume that the readers will take the time to review the protocol in OSF. Again, to minimize the cognitive overload, the authors should have this information in the manuscript.

We agree that key information from the OSF protocol should be clearly presented within the manuscript itself. We have now explicitly included the research question in the Background (lines 107–110). We also note that the registered project generated two related papers, one of which has already been published. The current manuscript addresses one specific component of the broader protocol. In line with PLOS ONE guidelines, the related published paper has been cited in this manuscript (see line 129) and was also uploaded as an additional file for peer review purposes to ensure full transparency.

There should be a paragraph at the end of the results synthesizing the findings. What does the result mean to the broader audience? How do they answer your research questions? The discussion recapped some of the results but needs to make a more concrete effort to connect them to the broader context of OHC and to offer recommendations for potential future research.

To avoid redundancy between the Results and Discussion sections, we integrated the requested synthesis at the beginning of the Discussion (see lines 383–392). We have also strengthened the Discussion to offer clearer implications and directions for future research. These revisions summarise the key findings, explains how they address the research question, and positions the results within the broader OHC context.

Regarding on the writing and mechanism, it is mostly in a good shape except for few typos and paragraph formatting. The authors will need to proof them before the next submission since PLOS One does not have a copyeditor who could clean up the manuscript before publishing.

We have carefully proofread the entire manuscript to correct typographical errors and improve paragraph formatting in preparation for the revised submission.
